# Towards Precision Nutrition: A Novel Smartphone-Connected Biosensor for Point-of-Care Detection of β-Hydroxybutyrate in Human Blood and Saliva

**DOI:** 10.3390/s25144336

**Published:** 2025-07-11

**Authors:** Cristina Tortolini, Massimiliano Caprio, Daniele Gianfrilli, Andrea Lenzi, Riccarda Antiochia

**Affiliations:** 1 Department of Experimental Medicine, Sapienza University of Rome, V.le Regina Elena 324, 00166 Rome, Italy; cristina.tortolini@uniroma1.it (C.T.); daniele.gianfrilli@uniroma1.it (D.G.); andrea.lenzi@uniroma1.it (A.L.); 2 Laboratory of Cardiovascular Endocrinology, IRCCS San Raffaele, 00163 Rome, Italy; massimiliano.caprio@uniroma5.it; 3 Department for the Promotion of Human Sciences and Quality of Life, San Raffaele Roma Open University, 00166 Rome, Italy; 4 Department of Chemistry and Drug Technologies, Sapienza University of Rome, P.le Aldo Moro 5, 00185 Rome, Italy

**Keywords:** β-hydroxybutyrate, chitosan nanoparticles, ferricyanide carbon screen printed electrode, smartphone connected biosensor, precision nutrition

## Abstract

Precision nutrition is an emerging approach that tailors dietary recommendations based on an individual’s unique genetic, metabolic, microbiome, and lifestyle factors. β-hydroxybutyrate (β-HB) is a key ketone body produced during fat metabolism, especially in states of fasting, low-carbohydrate intake, or prolonged exercise. Therefore, monitoring β-HB levels provides valuable insights into an individual’s metabolic state, making it an essential biomarker for precision and personalized nutrition. A smartphone-connected electrochemical biosensor for single-use, rapid, low-cost, accurate, and selective detection of β-HB in whole blood and saliva at the Point-of-Care (POC) is reported. A graphite screen-printed carbon electrode modified with potassium ferricyanide (Fe(III)GSPE) was used as an electrode platform for the deposition of β-hydroxybutyrate dehydrogenase (HBDH), nicotinamide adenine dinucleotide oxidized form (NAD^+^), and chitosan nanoparticles (ChitNPs). An outer poly(vinyl) chloride (PVC) diffusion-limiting membrane was used to protect the modified electrode. The biosensor showed a linear range in the clinically relevant range, between 0.4 and 8 mM, with a detection limit (LOD) of 0.1 mM. The biosensor was tested on human blood and saliva samples, and the results were compared to those obtained with a commercial ketone meter, showing excellent agreement.

## 1. Introduction

β-hydroxybutyrate (β-HB) is one of the main endogenous circulating ketone bodies (KBs), and under normal conditions its blood levels are <0.6 mM. During nutritional ketosis, its plasma levels range between 0.6 and 1.5 mM, whereas they are usually between 1.5 and 3 mM in hyperketonemia and above 3 mM under diabetic ketoacidosis (DKA) [1]. A ketosis state is observed in conditions of intense physical exercise, vomiting, or fasting and in type 1 diabetes mellitus patients with insulin deficiency [2]. β-HB is the primary form of KBs present in blood during the onset of ketosis and DKA. Therefore, accurate detection of b-HB levels in biological samples is very important for early diagnosis of diabetes and ketonemia. Moreover, for individuals following ketogenic or low-carb diets, tracking b-HB levels ensures optimal ketosis, preventing metabolic imbalances. Various methods have been developed to detect β-HB concentrations in blood [3], urine (via acetoacetate estimation) [4], and breath (via acetone detection) [5]. Spectrophotometry, chemiluminescence [6], gas chromatography–mass spectrometry (GC-MS) [7,8], and liquid chromatography–tandem mass spectrometry (LC–MS/MS) [9] have been largely reported in the literature. However, these methods are time-consuming and require special apparatuses, making them unsuitable for point-of-care (POC) detection.

Electrochemical biosensors may overcome these issues. Most of them reported in the literature for β-HB detection use HBDH (β-hydroxybutyrate dehydrogenase) enzyme involving the NAD^+^/NADH (nicotinamide adenine dinucleotide, oxidized and reduced forms) system, leading to the production of the electroactive detectable NADH. The inherent problems related to large overvoltage for the electrochemical oxidation of NADH at conventional solid electrodes have been minimized using different electrode materials [10]. However, the biosensors reported in the literature based on direct NADH detection led to electrode fouling, due to the adsorption of the NAD^+^ dimer reaction products, causing problems of low reusability and low stability of the biosensor. Therefore, most biosensors for β-HB detection reported in the literature are “second-generation” biosensors, based on the use of a redox mediator, which regenerates the enzymatically active NAD^+^ and is re-oxidized at the electrode surface at a suitable potential, where contributions of oxidizable blood and saliva constituents, such as ascorbic acid, uric acid, glucose, and oxygen, are avoided [11,12,13,14,15,16].

Another critical issue to be considered in the construction of the HBDH-based electrochemical platform is the efficient and stable confinement of NAD^+^/HBDH on the modified electrode surface. Some authors used CNTs [17], others use one or two layers of chitosan polymer [18,19,20] to facilitate the robust immobilization of HBDH/NAD^+^ biomolecules through the formation of covalent bonds. Therefore, most recent biosensors are fabricated through a complex process, where several layers are sequentially deposited on the electrode surface in order to optimize the anchoring of the mediator, cofactor, and enzyme, thus increasing the complexity of the system and costs, with a possible sensitivity loss over time.

This study aims to design a simple, one-layer, low-cost electrochemical biosensor for sensitive and selective detection of β-HB by using a portable potentiostat directly connected to a smartphone for POC signal reading. The main target of this work is to realize an extremely simple electrochemical platform by a single drop-casting step of a mixture of HBDH/NAD^+^/ChitNPs on a commercial Fe(III)GSPE. An outer PVC membrane was added as a protective and diffusion-limiting layer. The developed biosensor allowed the single-use detection of β-HB on both human blood and saliva samples, thus demonstrating the potential to fulfill the needs of a health precision nutrition and to move in the direction of the development of personalized medicine [21].

## 2. Materials and Methods

### 2.1. Reagents

The enzyme 3-hydroxybutyrate dehydrogenase (HBDH), at ~3 units/mg protein, from *Rhodobacter sphaeroides* was supplied by Roche. 3-hydroxybutyric acid (3-HB, 95%), free acid (NAD^+^), β-nicotinamide adenine dinucleotide, reduced disodium salt hydrate (NADH), chitosan (Chit, low-molecular-weight deacetylated chitin), sodium tripolyphosphate (TPP, 85%), glacial acetic acid, potassium ferricyanide (III) (K_3_[Fe(CN)_6_], 99.0%), potassium ferrocyanide (II) (K_4_[Fe(CN)_6_], 98.5–102.0%), poly(vinyl) chloride (PVC), tetrahydrofurane (THF), and a b-HB assay kit (MAK540-1KT) were obtained from Merck (Milan, Italy). A BHB ELISA kit was purchased from Abbexa Ltd. (Cambridge, UK). All solutions were prepared in 0.1 M TRIS and 0.1 M KCl, at a pH of 7.5. High-purity deionized water (resistance: 18.2 MΩ cm at 25 °C; TOC < 10 µg L^−1^) (Millipore, Molsheim, France) was used throughout all the experiments. ChitNPs were synthetized according to a method described in our previous work [22].

UV-Visible spectra were recorded on a SpectraMax M2e spectrophotometer (Molecular Devices, Wokingham, UK) with Polystyrene Macro Cuvettes (capacity 4 mL, Fisherbrand^TM^ Fisher Scientific Italia, Milan, Italy), operating at a resolution of 1 nm from 400 to 700 nm. The reading was carried out at 450 nm and 37 °C. The data were successively analyzed with the SoftMax Pro software 7.0.

### 2.2. Apparatus

Transmission electron microscopy (TEM) was performed on a JEOL 1200 EX2 (JEOL USA, Peabody, MA, USA) operating with an acceleration voltage of 150 kV. The size of the ChitNPs was determined using ImageJ software25 on at least 10 TEM images. Samples for TEM examination were prepared by drop-casting 10 μL of ChitNP solution onto carbon-coated copper grids (C200Cu, EMR Resolutions, Sheffield, UK), which left to dry for 24 h and then stained with 2% phosphate tungsten acid.

Cyclic voltammetry (CV) and differential pulse voltammetry (DPV) measurements were performed by a portable potentiostat (Sensit Smart potentiostat by PalmSens, Houten, The Netherlands) directly connected to a smartphone. The software used is PSTrace5. The experiments were carried out with a screen-printed electrode (SPE), consisting of a working electrode (a potassium ferricyanide-modified graphite SPE (DRP-110FERRI, d = 4 mm, Metrohm Italiana, Oreggio, Italy) (Fe(III)GSPE)), a silver pseudo-reference electrode, and a carbon rod auxiliary electrode. The ketone meter was a Wellion Galileo GLU/KET blood glucose and ketone meter, including strips.

### 2.3. Biosensor Fabrication

**Figure 1:**
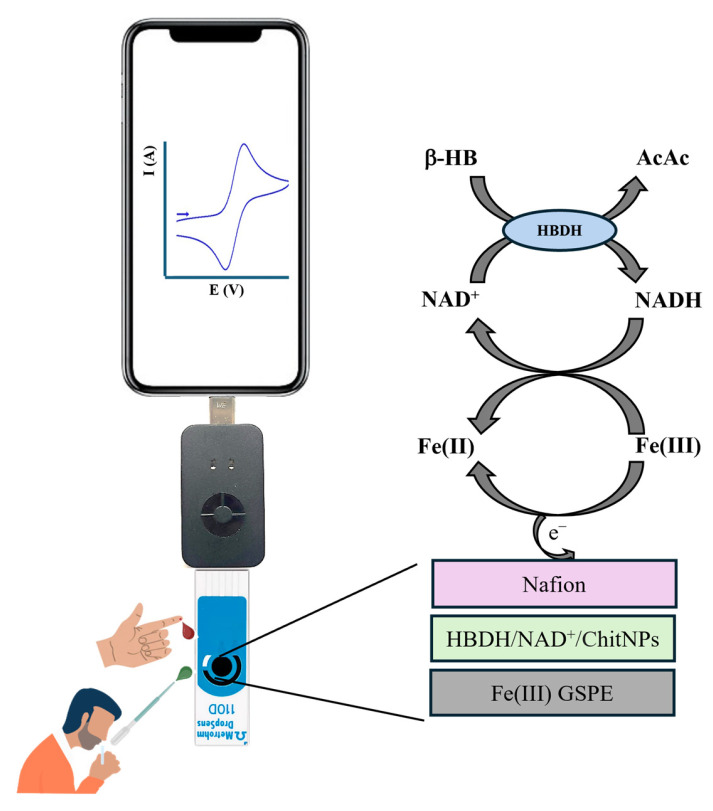
Schematic representation of the biosensor: electrode platform, mechanism, and use.

In total, 5 µL of a mixed solution of 10 mM NAD^+^, HBDH (15 U/mL in 0.1 M PBS, pH 7.5), and ChitNPs (1 mg mL^−1^) was drop-casted onto the Fe(III)GSPE and left to dry overnight at 4 °C. Then, 1 µL of PVC of a 2% THF solution was drop-casted and left to dry at RT. Figure 1 shows a schematic representation of the modified electrode.

### 2.4. Real Samples Analysis

One fingertip whole-blood droplet (≅10 μL), pricked with a one-shot medical needle, from three volunteers each was directly drop-casted onto the electrode surface or onto the ketone meter strip for DPV measurements and ketone meter reading, respectively. Human saliva samples from the same volunteers were collected in 3 mL tubes. One droplet (≅10 μL) was directly drop-casted onto the electrode surface for DPV analysis.

Both blood and saliva samples were analyzed at room temperature (25 °C), immediately after collection.

## 3. Results and Discussion

### 3.1. TEM Characterization

**Figure 2:**
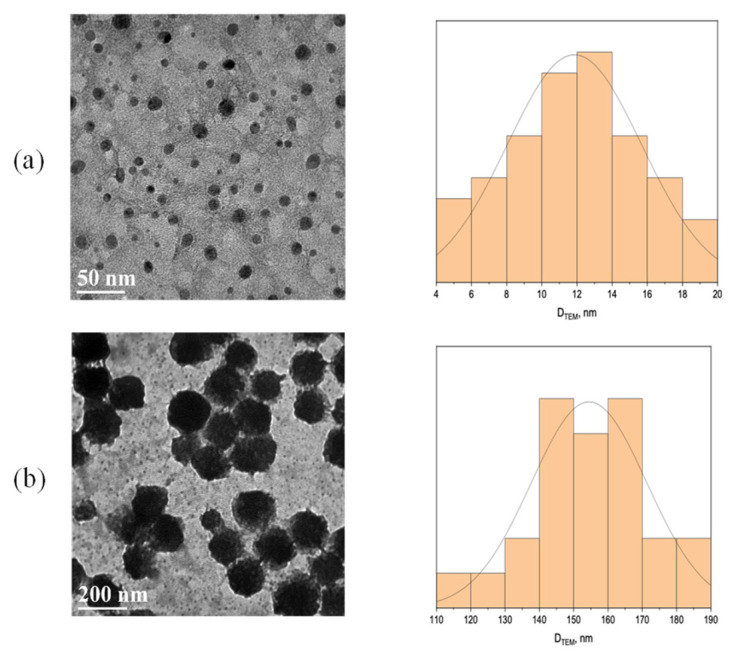
TEM images (**left**) and size distribution (**right**) of ChitNPs (**a**) and ChitNPs/HBDH/NAD^+^ (**b**).

TEM images revealed the amorphous nature of the ChitNPs, with a spherical form, homogeneous size distribution, and crystalline structure without aggregates and clusters. The average diameter turned to be 11.8 ± 3.8 nm (see Figure 2a). After the immobilization of enzymes and NAD^+^, an increase in particles size was observed, with a diameter of 154.5 ± 16.9 nm (Figure 2b), remaining within the nanoscale. A similar behavior has been already reported in the literature for ChitNPs and L-asparaginase [23].

### 3.2. Electrochemical Characterization of the PVC/HBDH/NAD^+^/ChitNPs/Fe(III)GSPE Platform

**Figure 3:**
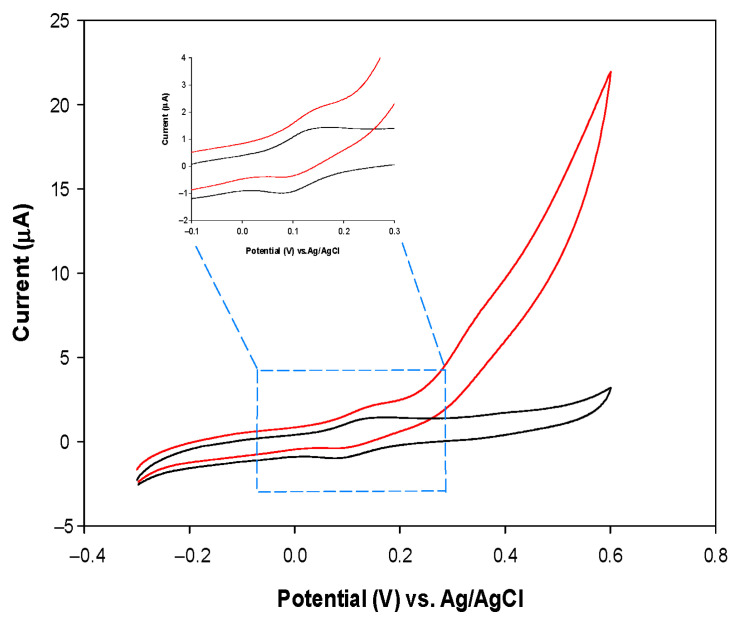
CVs of Fe(III)GSPE obtained in the absence (black) and presence (red) of 5 mM NADH. Inset: zoom in the range from −0.1 to 0.3 V. Experimental conditions: 0.1 M TRIS buffer and 0.1 M KCl, at pH 7.5 and at a scan rate of 10 mV/s.

Preliminary CV experiments were carried out with the Fe(III)GSPE electrode in the absence and presence of 5 mM NADH in order to check the electrocatalytic activity of the Fe(III)GSPE electrode towards NADH oxidation, due to electron shuttling from NADH by the Fe(III) mediator to the electrode surface (Figure 3). An increase in the anodic current with a slight reduction in the cathodic peak is observed after adding NADH, compared to the bare Fe(III)GSPE electrode, indicating that the catalytic process is occurring (Figure 3, inset). The oxidation peak corresponds to the oxidation of NADH at NAD^+^, due to electron shuttling by the Fe(III) mediator from NADH to the electron surface. These results demonstrate the catalytic ability of the Fe(III)GSPE electrode for NADH oxidation [24].

**Figure 4:**
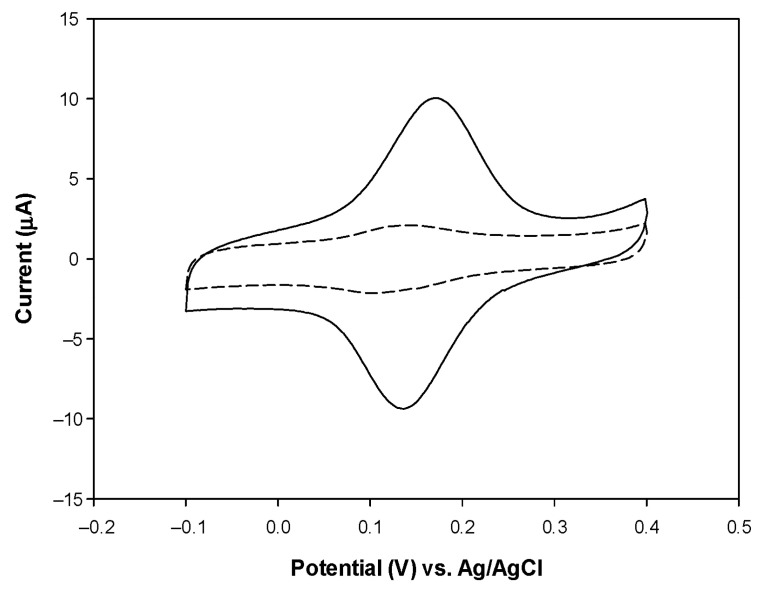
CVs of PVC/HBDH/NAD^+^/ChitNPs/Fe(III)GSPE biosensor in 0.1 M TRIS buffer, 0.1 M KCl, and NAD^+^ = 10 mM in absence (dashed line) and in presence (solid line) of β-HB = 3mM.

Next, CVs were recorded with the PVC/HBDH/NAD^+^/ChitNPs/Fe(III)GSPE biosensor in absence and presence of 3.0 mM β-HB, in order to demonstrate that the enzymatically generated NADH from the reaction between β-HB and NAD^+^, catalyzed by HBDH, effectively reaches the underlying Fe(III)SPE and undergoes electrocatalytic oxidation (Figure 4). It is releveant to note an increase in both the anodic and cathodic peak current after the addition of β-HB, with a slight anodic shift in the peak potentials, indicating that the enzyme is reacting with the analyte with the formation of NADH, which is then re-oxidized by the Fe(III)mediator. Such behavior is a fundamental prerequisite for the successful functioning of the β-HB biosensor. The shape of the voltammogram with the two peaks (Figure 4) demonstrates that the steady-state conditions are not achieved, probably because of a too low concentration of β-HB or due to the slow kinetics of the enzymatic system, which do not assure the constancy of the NADH concentration on the electrode surface.

### 3.3. Effect of NAD^+^ and pH on Biosensor Current Response

**Figure 5:**
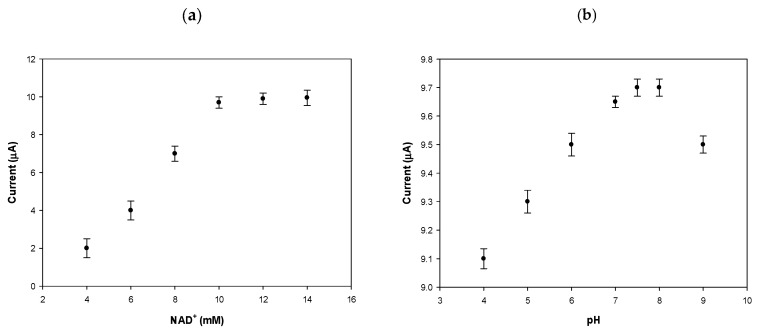
Effect of the concentration on current response of NAD^+^ (**a**) in 0.1 M TRIS buffer and 0.1 M KCl, at pH 7.5, with β-HB = 3 mM, and pH (**b**) in 0.1 M TRIS buffer and 0.1 M KCl, with NAD^+^ = 10 mM and β-HB = 3 mM.

In order to optimize the PVC/HBDH/NAD^+^/ChitNPs/Fe(III)GSPE biosensor response, the effect of NAD^+^ concentration and pH solution were investigated. Figure 5a shows the dependence of the current responses on the cofactor concentrations, when NAD^+^ was varied between 4 and 14 mM with a fixed amount of HBDH (15 U/mL) on the electrode surface, with β-HB added to the solution (3 mM). The optimal NAD^+^ concentration turned to be 10 mM. The NAD^+^ concentration cannot be too low to ensure that it would not be the rate-limiting factor in the enzymatic reaction between β-HB and NAD^+^, which would determine a current decrease in the biosensor response. On the other hand, excess NAD^+^ would increase the cost of the biosensor. Therefore, a concentration of 10 mM NAD^+^ was used in further experiments.

The effect of pH was studied in the range 4.0–9.0. The results are shown in Figure 5b, with an optimum pH response obtained at pH 7.5 in TRIS buffer, close to physiological conditions.

### 3.4. Detection of β-HB with PVC/HBDH/NAD^+^/ChitNPs/Fe(III)GSPE Biosensor

**Figure 6:**
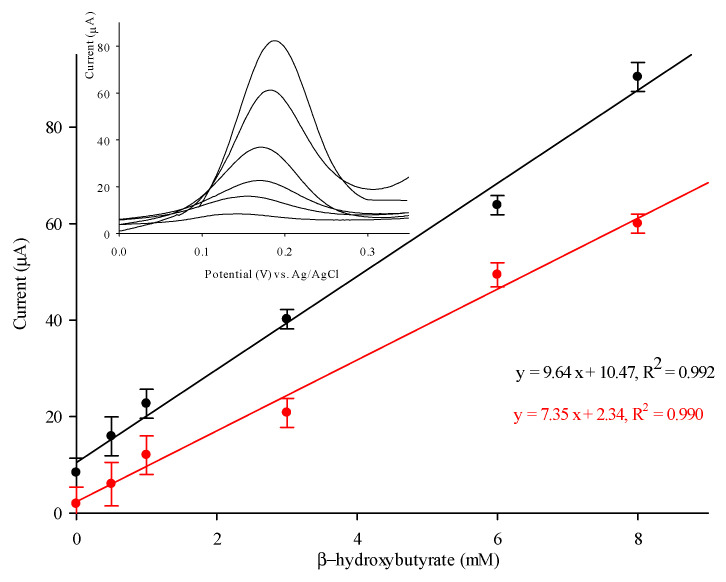
Calibration curve of the β-HB biosensor with ChitNPs (black curve) and pristine Chit (red curve). Inset: DPV curves of ChitNP-based biosensor.

Figure 6 shows the calibration curve and the DPV responses (inset) obtained with the biosensor at increasing β-HB concentrations. The calibration plot (black curve) exhibits a good linear relation (y = 9.64x + 10.47, where y represents the current in μA, and x is the concentration of the β-HB solution in mM) in an extended linear range between 0.4 and 8 mM, with a correlation coefficient of 0.992. The current reaches saturation above 10 mM. The detection limit (LOD) is estimated to be 0.1 mM, estimated on the 3 S_d_/m criteria, where S_d_ represents the standard deviation of the background signal and m the slope of the calibration plot, with the sensitivity amounting to 0.79 μA cm^−2^ mM^−1^. Figure 6 (red curve) shows the calibration curve obtained with a similar biosensor constructed by using pristine chitosan (Chit) instead of ChitNPs, showing lower sensitivity (0.66 μA cm^−2^ mM^−1^). By introducing ChitNPs instead of pristine Chit onto the modified electrode, an enhanced response for β-HB was observed. Chitosan material, largely employed to enhance sensor’s sensitivity, converted to a nano size, definitely assists in improving properties. The HBDH/NAD^+^, immobilized into the porous structure of the ChitNPs through adsorption, may enable close proximity between sensing components, which promotes faster electron transfer from β-HB to the electrode surface [19].

The apparent Michaelis–Menten constant for the PVC/HBDH/NAD^+^/ChitNPs/Fe(III)GSPE biosensor was calculated to be 5.8 mM from the Lineweaver–Burk equation, showing the high affinity of HBDH, immobilized with NAD^+^ and ChitNPs, for the substrate β-HB, thus attesting to the high biocompatibility of the electrochemical platform. The K_M_ value obtained is in agreement with the values reported in the literature for similar systems [15].

Table 1 shows a comparison with all other biosensors for β-HB detection reported in the literature. It can be easily noted that the proposed biosensor offers a combination of the most extended linear range/lower detection limit, which is of extreme importance for its potential application in clinical settings, where the linear range must be sufficiently large and within the clinically relevant range of the analyte, maintaining, at the same time, a low LOD and a good sensitivity.

### 3.5. Reproducibility, Stability, and Selectivity

The reproducibility of our biosensor was calculated to be 2.3 and 4.8 % (*n* = 5), in terms of the relative standard deviation (RSD) of the slopes and the intercept of the calibration curves obtained with five (*n* = 5) different electrodes, modified in exactly the same way. The RSD values obtained confirmed the good reproducibility of the proposed system.

**Figure 7:**
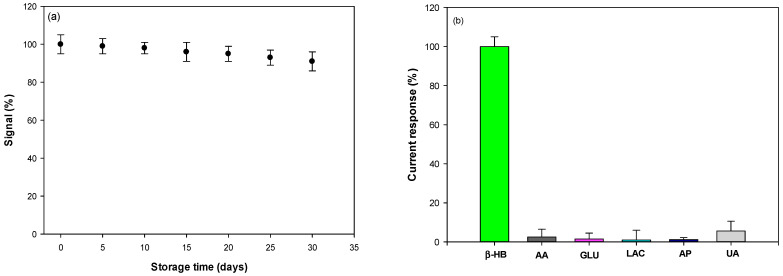
Stability (**a**) and selectivity (**b**) of the PVC/HBDH/NAD^+^/ChitNPs/Fe(III)GSPE biosensor. AA = ascorbic acid; GLU = glucose; LAC = lactate; AP = acetaminophen; UA = uric acid; AC = acetone; AcAc = acetoacetic acid.

Then, the stability of the β-HB biosensor was determined by testing the DPV responses of the proposed biosensor, stored dry at 4 °C for 1 month and monitored at regular intervals of 5 days. As a result, the biosensor was quite stable, maintaining >98% of its initial response after 10 days and >90% after 1 month (Figure 7a). The excellent stability is probably due to the bio-nanocomposite including ChitNPs, enzymes, and NAD^+^, which avoid enzyme denaturation, allowing the maintenance of good catalytic properties.

Lastly, the selectivity of the proposed biosensor was assessed by studying the effects of the most common interferent compounds present in blood and saliva. In particular, DPV responses were recorded in a solution containing 500 μM of β-HB, ascorbic acid (AA), glucose (GLU), lactate (LAC), acetaminophen (AP), uric acid (UA), acetone (AC), and acetoacetic acid (AcAc). The results are reported in Figure 7b and show that the relative responses obtained for all compounds tested were weak under the testing conditions. In particular, the responses to ascorbic acid and uric acid, which are the interferents present in higher amounts in biological samples, were only 2.5% and 5.6% when the β-HB response was taken as 100%, attesting to the high selectivity of the biosensor.

Moreover, 500 μM of cholesterol and the amino-acids L-leucine, L-lysine, L-valine, L-isoleucine, L-methionine, L-threonine, L-glutamine, and L-glutamate have been tested and no significant current signal occurred, attesting no interference from these substances.

The high selectivity of the biosensor is also assured by the PVC permselective outer membrane deposited on top of the modified electrode [27].

### 3.6. Detection of β-HB in Human Blood and Saliva

The proposed biosensor was tested in the human capillary whole blood of three volunteers, one following a normal diet and two following a very-low-calorie ketogenic diet. The results are reported in Table 2, with a comparison with those obtained on the same individuals with a commercial ketone meter [28] and a conventional ELISA reference method. They showed excellent agreement with both methods (% difference ≤ 10%), with slightly lower values recorded with the biosensor, as the red cells in capillary whole blood may hinder the diffusion of β-HB onto the electrode surface, thus lowering the current response. It is interesting to note that the standard deviation values obtained with our biosensor are lower than those obtained with the commercial ketone meter (Table 2) [29].

**Figure 8:**
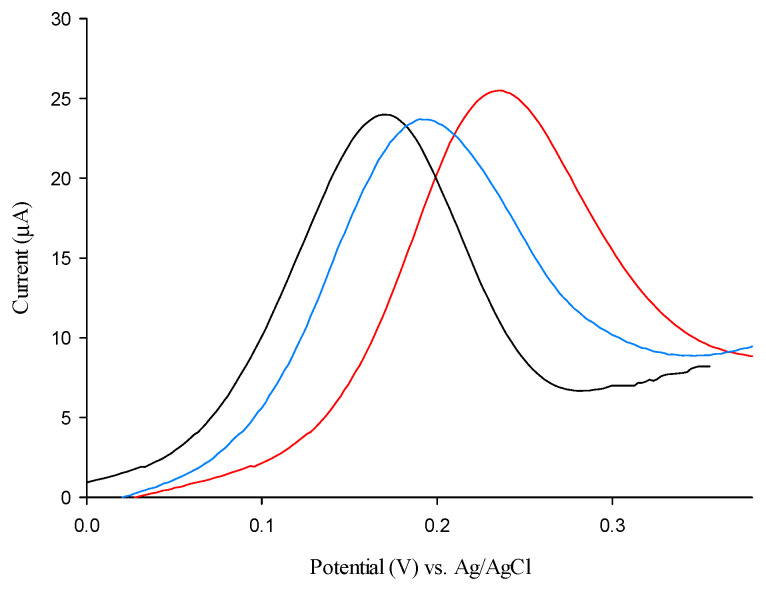
DPV curves of 1 mM β-HB (black curve), human sample 2 saliva (blue curve), and human sample 2 blood (red curve).

The biosensor also allowed the non-invasive detection of β-HB in human saliva (Figure 8, Table 2), where β-HB concentration is known to be slightly lower but with similar temporal trends [19]. A small anodic shift in peak potential is registered for saliva (Figure 8, blue curve) and blood (Figure 8, red curve) samples, compared to PBS (Figure 8, black curve). In this case, the results obtained with the biosensor were compared only to those obtained with the ELISA test (Table 2), showing good agreement between the two methods (RSD% values < 12), indicating a satisfactory accuracy, thus suggesting the possible use of the proposed biosensor in the assay of β-HB in both human capillary blood and saliva samples.

## 4. Conclusions

A disposable PVC/HBDH/NAD^+^/ChitNPs/Fe(III)GSPE biosensor was demonstrated to successfully work for POC β-HB detection in both human blood and saliva. It shows favorable analytical features, including an extended linear range in the clinically relevant β-HB range, high sensitivity, good reproducibility, and good stability, which, together with its ease of use and short response time (20 s), make the proposed biosensor a suitable device for β-HB measurements that can be easily used by both patients and health care professionals. The low cost of preparation of the biosensor is another key aspect of this device. The cost of a single screen-printed electrode after the proposed modification is calculated to be < 3 EUR, cheaper than the cost of a single strip used for measurements with the commercial ketone meter employed in this study (……EUR). Moreover, the smart phone-connected biosensor allows patients to upload and store the data, essential for tight nutrition tracking, thus enabling the nutritionist to customize a personalized, effective, and sustainable diet, which is the main goal of a precision nutrition approach. Lastly, the biosensor has also been successfully tested for β-HB detection in saliva, allowing safe and non-invasive testing compared to the discomfort of finger pricks in blood analysis.

## Figures and Tables

**Table 1:** Comparison of biosensor performances with other recent β-HB biosensors reported in the literature.

Electrode Platform	Electrochemical Signal	Sensing Modalities	Linear Range (mM)	LOD (µM)	Test Samples	Ref.
Iridium–carbon ink/HBDH/NAD^+^/BSA	NADH oxidation	Amperometry	0–10	-	Bovine serum	[25]
CarbonSPE/K_3_[Fe(CN)6]/HBDH/NAD^+^	Mediator oxidation	Amperometry	0.01–5.28	14.41	Human blood and serum	[12]
Graphene nanosheets/CarbonSPE/ferro-ferricyanide/HBDH/NAD^+^/glycerol	Ferrocyanide oxidation	CV	0.00001–0.1 0.25–3.0	0.0002	Spiked bovine serum	[26]
Carbon SPE/RGO/thionine/HBDH/NAD^+^	Thionine oxidation	Amperometry	0.01–1	9	Spiked human serum	[14]
Carbon ink/Fe(III)-Fe(II)/BHDH/NAD^+^	Fe(II) oxidation	CA	0–4	-	Human blood	[13]
Au coated/carbon SPE/TBO/HBDH/NAD^+^/ MWCNTs/Chit/PVC	TBO oxidation	Amperometry	0.1–1 1–3	65	Human saliva	[19]
Au/GA/BSA/HBDH/NAD+	NADH oxidation	CV	0.6–1	600	Human urine	[4]
GSPE/Ru(II)/GO/HBDH/NAD^+^	Ru(II) oxidation	Amperometry	0.2–2.0	-	Bovine serum	[16]
Fe(III)GSPE/HBDH/NAD^+^/ ChitNPs/PVC	Fe(II) oxidation	DPV	0.4–8	0.2	Human blood and saliva	this work

TBO = toluidine blue; MWCNTs = multi-walled carbon nanotubes; Chit = chitosan; BSA = bovin serum albumin; GA = glutaraldehyde; GO = graphene oxide; RGO = reduced graphene oxide; Ru(II) = ruthenium(II).

**Table 2:** Comparison of β-HB detection with the proposed biosensor in whole blood and saliva with a commercial ketone meter and a conventional ELISA reference method.

Human Sample	Human Blood and Saliva b-HB Concentration (mM)				
	Biosensor * (Blood)	Ketone Meter * (Blood)	Biosensor * (Saliva)	ELISA (Blood)	ELISA (Saliva)
1	0.2 ± 0.1	0.2 ± 0.2	0.1 ± 0.1	0.1 ± 0.1	0.1 ± 0.1
2	1.1 ± 0.2	1.2 ± 0.3	1.0 ± 0.2	1.1 ± 0.2	0.9 ± 0.1
3	1.6 ± 0.2	1.7 ± 0.3	1.5 ± 0.2	1.7 ± 0.2	1.4 ± 0.2

* Every test for each patient was repeated 3 times (N = 3). The value reported in Table 2 is the average value.

## Data Availability

Data are contained within the article.
